# Supplementary material for: colocr: an R package for conducting co-localization analysis on fluorescence microscopy images
Source: PeerJ. 2019 Jul 4;7:e7255. doi: 10.7717/peerj.7255 (PMC6612416; doi:10.7717/peerj.7255)
Supplement: Supplemental Information 1 [file peerj-07-7255-s001.doc]

# load required libraries

library(imager)

library(colocr)

library(tidyverse)

library(xtable)

library(cowplot)

# get image path

fl <- system.file('extdata', 'Image0003_.jpg', package = 'colocr')

# load images and channels

img <- image_load(fl)

img1 <- channel(img, 1)

img2 <- channel(img, 2)

# generate figure of images and channels

par(mfrow = c(1,3), mar = c(0, 0, 1, 0))

plot(img, axes = FALSE, main = 'Merge')

plot(img1, axes = FALSE, main = 'Channel One')

plot(img2, axes = FALSE, main = 'Channel Two')

# generate manuscript figure1

figure1 <- list(

p_a = function() plot(img, axes = FALSE),

p_b = function() plot(img1, axes = FALSE),

p_c = function() plot(img2, axes = FALSE)

)

plot_grid(plotlist = map(figure1, ggdraw),

nrow = 1,

scale = 1.1,

labels = 'AUTO',

label_size = 10,

label_fontface = 'plain') %>%

ggsave(filename = 'figure1.png',

width = 18, height = 6, units = 'cm')

# select regions of interest

par(mfrow = c(2,2), mar = c(0, 0, 1, 0))

img %>%

roi_select(threshold = 90,

shrink = 10,

fill = 5,

clean = 10,

n = 3) %>%

roi_show()

# generate manuscript figure2

img_roi <- img %>%

roi_select(threshold = 90,

shrink = 10,

fill = 5,

clean = 10,

n = 3)

labels <- attr(img_roi, 'label')

dims <- dim(grayscale(img_roi))

a <- array(labels, dim = dims)

px <- cimg(a)

img1 <- channel(img_roi, 1)

img2 <- channel(img_roi, 2)

figure2 <- list(

p1 = function() plot(img, axes = FALSE),

p2 = function() plot(px, axes = FALSE),

p3 = function() {plot(img1, axes = FALSE); highlight(px)},

p4 = function() {plot(img2, axes = FALSE); highlight(px)}

)

plot_grid(plotlist = map(figure2, ggdraw),

nrow = 2,

scale = 1.1,

labels = 'AUTO',

label_size = 10,

label_fontface = 'plain') %>%

ggsave(filename = 'figure2.png',

width = 18, height = 18, units = 'cm')

# check pixel intensities

par(mfrow = c(1,2), mar = c(4, 4, 1, 1))

img %>%

roi_select(threshold = 90,

shrink = 10,

fill = 5,

clean = 10,

n = 3) %>%

roi_check()

# generate manuscript figure3

par(mar = c(10,10,10,10))

pix_int <- .intensity_get(img_roi)

d1 <- density(pix_int[[1]])

d2 <- density(pix_int[[2]])

xlim <- c(min(c(d1$x, d2$x)), max(c(d1$x, d2$x)))

ylim <- c(min(c(d1$y, d2$y)), max(c(d1$y, d2$y)))

figure3 <- list(

p1 = function() {

par(mar=c(9,9, 1, 1))

plot(pix_int[[1]], pix_int[[2]],

col = alpha(pix_int[[3]], 0.3),

pch = 16,

xlab = 'Channel One', ylab = 'Channel Two',

cex = .5,

cex.lab = 1.3)

},

p2 = function() {

par(mar=c(9,9, 1, 1))

plot(d1$x, d1$y,

xlim = xlim, ylim = ylim,

type = 'l', col = alpha('darkgreen', .5),

xlab = 'Pixel Value', ylab = 'Density',

cex.lab = 1.3,lwd = 1.5)

lines(d2$x, d2$y,

col = alpha('magenta', .5),

lwd = 1.5)

}

)

plot_grid(plotlist = figure3,

scale = .9,

nrow = 1,

labels = 'AUTO',

label_size = 10,

label_fontface = 'plain') %>%

ggsave(filename = 'figure3.png',

width = 20, height = 10, units = 'cm')

# calculate co-localization stats

img %>%

roi_select(threshold = 90,

shrink = 10,

fill = 5,

clean = 10,

n = 3) %>%

roi_test(type = 'both')

# generate table for co-colocalization stats

img %>%

roi_select(threshold = 90,

shrink = 10,

fill = 5,

clean = 10,

n = 3) %>%

roi_test(type = 'both') %>%

mutate(roi = row_number()) %>%

rbind(c(mean(.$pcc), mean(.$moc), 'Average')) %>%

select(roi, everything()) %>%

mutate_at(vars(pcc, moc), function(x) round(as.numeric(x),2)) %>%

setNames(c('ROI', 'PCC', 'MOC')) %>%

xtable(caption = '\\textbf{Co-localization statistics.}',

align = 'cccc',

label = 'tab:table2') %>%

print(include.rownames = FALSE,

booktabs = TRUE,

add.to.row = list(pos = list(3),

command = '\\midrule '),

caption.placement = 'top',

table.placement = 'H',

sanitize.text.function = identity,

comment = FALSE,

file = 'table2.tex')

# copy source code to manuscript dir

file.copy('script.R',

to = './script.R')
